# Growth Prediction of the Total Bacterial Count in Freshly Squeezed Strawberry Juice during Cold Storage Using Electronic Nose and Electronic Tongue

**DOI:** 10.3390/s22218205

**Published:** 2022-10-26

**Authors:** Jing-Wen Zhang, Lei-Qing Pan, Kang Tu

**Affiliations:** College of Food Science and Technology, Nanjing Agricultural University, Nanjing 210095, China

**Keywords:** total bacterial count, freshly squeezed strawberry juice, E-nose, E-tongue, predictive microbiology

## Abstract

The growth models of total bacterial count in freshly squeezed strawberry juice were established by gas and taste sensors in this paper. By selecting the optimal sensors and fusing the response values, the Modified Gompertz, Logistic, Huang and Baranyi models were used to predict and simulate the growth of bacteria. The results showed that the R^2^ values for fitting the growth model of total bacterial count of the sensor S7 (an electronic nose sensor), of sweetness and of the principal components scores were 0.890–0.944, 0.861–0.885 and 0.954–0.964, respectively. The correlation coefficients, or R-values, between models fitted by the response values and total bacterial count ranged from 0.815 to 0.999. A single system of electronic nose (E-nose) or electronic tongue (E-tongue) sensors could be used to predict the total bacterial count in freshly squeezed strawberry juice during cold storage, while the higher rate was gained by the combination of these two systems. The fusion of E-nose and E-tongue had the best fitting-precision in predicting the total bacterial count in freshly squeezed strawberry juice during cold storage. This study proved that it was feasible to predict the growth of bacteria in freshly squeezed strawberry juice using E-nose and E-tongue sensors.

## 1. Introduction

Strawberry (*Fragaria × ananassa* Duch.) is widely planted in the world. Strawberry juice is a processed product loved by consumers because of its rich flavor, nutrients and bioactive substances [1]. However, microorganisms will grow and reproduce with the help of food matrix, such as carbohydrate, protein and fat. Microbial contamination is an important concern for juice industry and microbial metabolites will affect the flavor and taste of freshly squeezed strawberry juice, reducing its economic value during cold storage.

Thermal processing treatment can reduce the growth of microorganisms to prolong the shelf life effectively. However, it can cause the loss of nutrients and change the flavor and appearance of products [2]. Compared with commercially sterilized strawberry juice, mechanically squeezing fruit juice can retain its nutrients, flavor and taste to the greatest extent.

At present, there is no unified national standard for evaluating the nutrition, quality and safety of freshly squeezed strawberry juice. The local standard of the Zhejiang Province (China) points out that the safety will be lost when the total bacterial count reaches 5 Log(CFU/mL) (CFU: Colony Forming Unit). Many methodologies have been developed to ensure the quality control of fruit juice. The traditional approach of plate counting is widely used in food microbial safety detection, but the operation is complex and time-consuming [3]. Molecular procedures, such as Polymerase Chain Reaction (PCR), real-time Polymerase Chain Reaction [4], Restriction Fragment Length Polymorphism (RFLP), ribosomal RNA sequencing [5] and DNA probes [6] are also applied. These techniques are efficient and sensitive, and can provide more information. However, the operations are complex and destructive, and the reagents used may be harmful to consumers’ health and the environment. At present, many studies use the impacts of the metabolites produced by microorganisms to study the microbial contamination in foodstuff. For example, microbial volatile organic compounds (MVOCs) produced from metabolic activity and interaction with other microorganisms [7], as indicators of microbial growth, were applied in food safety inspection [8]. Ragaert, P. et al. studied the metabolic activity of yeasts in strawberry agar stored at 7 °C by the concentration of MVOCs and the yeasts’ sugar consumption [9]. Nieminen, T. et al. studied the volatile organic compounds (VOCs) to detect the fungal growth in strawberry jam [10]. Rojas-Flores, C. et al. explored whether the CO_2_ and VOCs of fungi can allow for the early detection of anthracnose and soft rot diseases in strawberry fruit during cold storage [11].

Predictive microbiology has been applied in the food and food inspections industries [12]. As a scientific-based tool, it evaluates the microbiological relations under certain environmental variables by applying and developing mathematic models [13], which can be classified into kinetic and probability models [14], empirical and mechanistic models [15] and primary, secondary and tertiary models [16] by the variables for modeling. For instance, the growth of *Proteus mirabilis* [17], *Pseudomonas* spp. [18] and *Salmonella* [19] in chicken were modeled and predicted under different temperatures. The growth model of *Pseudomonas* spp. [20], *Aeromonas* spp. and *Listeria monocytogenes* [21] in pork were performed. This tool was also applied on beef [22,23], vegetables [24,25,26], fruit [27] and so on. At present, many studies have completed rapid, accurate and non-destructive fitting of colony growth models by using hyperspectral imaging (HSI), an electronic nose (E-nose), etc., according to the optical and odor characteristics caused by the growth of microorganisms. Zhou et al. constructed the growth model of *P. fluorescens* in pork using HSI with the R^2^ ranging from 0.849–0.974 [6]. Achata, EM et al. predicted the growth of total viable count (TVC) on beef Longissimus dorsi muscle stored at 4 °C and 10 °C using HSI and chemometrics [28]. Tao et al. investigated the contamination of *Escherichia coli* (*E. coli*) in pork meat with R^2^ at 0.939 [29]. Zheng et al. performed the real-time prediction of TVC using HSI in pork during refrigerated storage and obtained the best model by the SVR with second derivation with R_p_ at 0.94. [30]. Gu et al. completed the growth prediction of *Pseudomonas aeruginosa* on agar plates and meat stored at 4 °C and 10 °C by E-nose sensing, and selected optimal sensors to fit the growth with higher correlation coefficients [31]. Tirnson, K et al. constructed a BPNN prediction model of the total bacterial count in chicken with R^2^ at 0.94 using an E-nose to evaluate the meat’s freshness [32]. Han et al. evaluated the freshness of fish stored at 4 °C quantitatively by E-nose and E-tongue with three-layer radial basis function neural network (RBF-NN) models [33]. Qiu et al. applied E-nose and E-tongue technology to the qualification and quantization of processed strawberry juice. The E-tongue system was shown to provide a better to qualitative discrimination and prediction than the E-nose among four kinds of different processed strawberry juices [34]. Meanwhile, strawberry juice under different processing approaches could be discriminated and characterized by E-tongue sensing with higher accuracy than E-nose by LDA, PLSR, RF and SVM [35]. The simultaneous utilization of these two systems could predict the quality of strawberry juice with higher accuracy.

There existed definite relationship between microbial contamination and the features of aroma and taste. E-tongue is capable of performing qualitative and quantitative analysis in the food safety index [36]. Aroma and taste features change in accordance with the deterioration of juice quality. In this study, we constructed the predictive growth model of total bacterial count respectively by use of the E-nose, E-tongue and the fusion information of the two systems, and compared the models to find a better way to predict the shelf life of freshly squeezed strawberry juice quickly and accurately.

## 2. Materials and Methods

### 2.1. Chemicals

Analytical reagents required for E-tongue detection were obtained from Sinopharm Chemical Reagent Co., Ltd. (Shanghai, China) as follows: potassium chloride, silver chloride, tartaric acid, ethanol, hydrochloric acid and potassium hydroxide.

### 2.2. Preparation of Freshly Squeezed Strawberry Juice

“Hong Yan” strawberries of the same maturity level (80% mature) were harvested in the Suoshi village, Nanjing, Jiangsu province, China. They were transported to the laboratory within 1 h to dissipate the field heat. Strawberries without mechanical damage were selected and the sepals were removed. They were squeezed using a juicer (SJ109-200, SUPOR Co., Ltd., Hangzhou, China) and filtered with two layers of 120-mesh gauze. Freshly squeezed strawberry juice was put into 50 mL test tubes that had been sterilized at 121 °C for 15 min, and then stored at a temperature of 4 ± 1 °C and humidity of 95% for 7 days.

The total bacterial count was determined every 12 h, and the aroma and taste characteristics of freshly squeezed strawberry juice were measured with E-nose and E-tongue sensors, respectively.

### 2.3. Determination of Total Bacterial Count of Freshly Squeezed Strawberry Juice during Cold Storage by Plate Counting

According to the national standard “Food Safety National Standard Microbiological Examination of Food—Determination of Total Bacterial Count” (GB 4789.2-2016), 25 mL of freshly squeezed strawberry juice was taken into a 225 mL triangular conical flask. Gradient dilution was carried out and two plates were set for each gradient. Ten repetitions were set at each storage time. The average values were used to fit the growth curve of the total bacterial count in freshly squeezed strawberry juice during cold storage.

### 2.4. Acquisition of E-Nose Information

An E-nose device (Airsense Analytics GmBh, Schwerin, Germany) is equipped with 10 sensors, each of which is sensitive to specific substances. The response values are related to the intensity of VOCs. The conditions of the E-nose were modified based on the Qiu et al. [37] as follows: Flow rate was set at 150 mL/min. Measurement time was set at 90 s in order to obtain stable responses. Gas flushing time was set at 60 s to eliminate interference between samples. Sample preparation time was 5 s, and automatic zeroing time was 5 s.

5 mL of freshly squeezed strawberry juice was drawn into 250 mL glass beakers sealed with tin foil. Samples were then kept at room temperature for 20 min to balance the headspace gas. Twenty samples were selected for each experiment. Fifteen samples were selected as a training dataset and five samples as a testing dataset. Response values of sensors after 70 s were stable, thus, values at 80 s were recorded for subsequent analysis.

### 2.5. Acquisition of E-Tongue Information

An E-tongue device (TS-5000Z, Insent, Atsugi, Japan) is composed of 6 basic taste sensor electrodes, including freshness, saltiness, sourness, bitterness, astringency and sweetness.

Measurement parameters were based on the research of Tian et al. [38]. Measurement time was set to 120 s and rinsing time of sensors was 10 s for each detection. A total of 60 mL of filtered freshly squeezed strawberry juice was used for E-tongue analysis with a TS-5000 taste system. Response values of sweetness were recorded 5 times, and the other taste information was collected 4 times. Each sample of different storage times was set to 20 repetitions.

### 2.6. Data Analyzing Methods

Growth curves of total bacterial count in freshly squeezed strawberry juice were fitted with MATLAB (R2015b, MathWork Inc., Natick, MA, USA). ANOVA and Pearson correlation analyses between response values of sensors and plate-counting results were completed using SPSS 18.0 software (IBM Corp., Armonk, NY, USA). Significant difference was analyzed through the Duncan method (*p* < 0.05). Graphs regarding the growth curves were drawn by Origin 2022.

### 2.7. Growth Curve Fitting

Data from traditional plate counting of total bacteria count and the response values of E-nose and E-tongue sensors were plotted to fit with the growth curve [6,31]. The Modified Gompertz, Logistic, Huang and Baranyi models were used to simulate the growth of total bacteria in freshly squeezed strawberry juice.

Modified Gompertz model:

$$N\left(t\right)=N_{0}+\left(N_{max}-N_{0}\right)\times e^{-e^{\left[\frac{\mu_{max}\times 2.718}{N_{max}-N0}\times \left(\lambda -t\right)+1\right]}}$$

Logistic model:

$$N\left(t\right)=N_{0}+\frac{N_{max}-N_{0}}{1+e^{\mu_{max}\times \left(\lambda -t\right)}}$$

Huang model:

$$N\left(t\right)=N_{0}+N_{max}-ln[e^{N_{0}}+\left(e^{N_{max}}-e^{N_{0}}\right)\times e^{-\mu_{max}\times \left(t+\frac{1}{4}\times ln\frac{1+e^{-4\times \left(t-\lambda \right)}}{1+e^{4\times \lambda \;}}\right)}$$

Baranyi model:

$$N\left(t\right)=N_{0}+\mu_{max}\times \left[t+\frac{1}{\mu_{max}}\times ln\left(e^{-\mu_{max}\times t+e^{-\lambda \times \mu_{max}}-e^{-\mu_{max}\times t-\lambda \times \mu_{max}}}\right)-\;\;\;\;\;ln\left(1+\frac{e^{\mu_{max}\times t+\frac{1}{\mu_{max}}\times ln\left(e^{-\mu_{max}\times t+e^{-\lambda \times \mu_{max}}-e^{-\mu_{max}\times t-\lambda \times \mu_{max}}}-1\right)}}{e^{N_{max}-N_{0}}}\right)\right]\;$$

For these four growth models, ***N*_0_**: initial total bacterial count or response values of E-nose and E-tongue; ***N_max_***: maximum total bacterial count or respond values of E-nose and E-tongue; ***μ_max_***: maximum specific growth rate (h^−1^); ***λ***: lag time (h).

Root mean square errors (RMSE) and coefficient of determination (***R*^2^**) were used to evaluate the fitting of these growth models [39]. High values of ***R*^2^** and low values of RMSE represent a good model [40].

$$\mathrm{RMSE}:\;RMSE=\sqrt{\frac{\sum_{i=1}^{n}{\left(y_{i}-y_{p}\right)}^{2}}{n-p}}$$

$$R^{2}:\;R^{2}=1-\frac{\left(n-1\right)}{\left(n-p\right)}\left(\frac{\sum_{i=1}^{n}{\left(y_{i}-y_{p}\right)}^{2}}{\sum_{i=1}^{n}{\left(y_{i}-y_{m}\right)}^{2}}\right)$$

***n***: the number of observed data points; *p*: the number of parameters; ***y_i_***: the measured values (LogCFU/mL); ***y_p_***: the predicted values (LogCFU/mL); ***y_m_***: the mean values (LogCFU/mL).

## 3. Results and Discussions

### 3.1. Growth Simulation of Total Bacterial Count by Traditional Plate-Counting Methods

**Figure 1:**
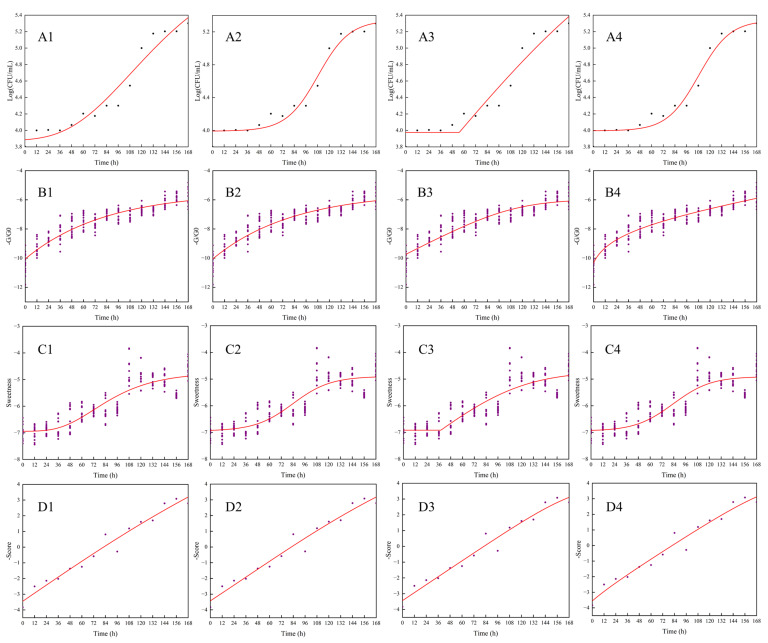
Fitting of the Modified Gompertz, Logistic, Huang and Baranyi models to the total bacterial count (**A1**–**A4**), the response values of S7 (**B1**–**B4**), sweetness (**C1**–**C4**) and the scores of PC1–PC4 extracted from E-nose and E-tongue readings (**D1**–**D4**) for freshly squeezed strawberry juice during cold storage.

The data obtained from plate counting was used for fitting the growth curve of the Modified Gompertz, Logistic, Huang and Baranyi models. As shown in Figure 1(A1–A4), the logarithm of total bacterial count (LogCFU/mL) increased nonlinearly with storage time. Growth of total bacterial count in freshly squeezed strawberry juice was slow at 4 °C with a long period of lag phase. Low temperature and the ratio of sugar to acid could, to some extent, inhibit the activity of bacterial growth in juice [41].

Different kinetic-fitting models and parameters of total bacterial count in freshly squeezed strawberry juice during cold storage at 4 °C are shown in Table 1. The fitting of the Logistic and Baranyi models, both with ***R*^2^** at 0.971, were better than those of the Modified Gompertz and Huang models. At the same time, the lag time of the total bacterial count in freshly squeezed strawberry juice fitted by these two dynamic models was longer. Low temperature (4 ± 1 °C) may inhibit the growth of microorganisms. They could adapt to the new condition during the longer lag time and then grow quickly. Among these four growth models, the simulated values of the total bacterial count by Logistic and Baranyi models were close with each other. ***R*^2^** ranged from 0.944–0.971, while RMSE was 0.103–0.143. A good model is obtained when ***R*^2^** is closer to 1 and the RMSE value is smaller. These four growth models could be used to stimulate the growth of the total bacterial count in freshly squeezed strawberry juice, although the fitting of Logistic and Baranyi models were strongest.

### 3.2. E-Nose Testing Results

#### 3.2.1. Gas Sensor Selection

**Figure 2:**
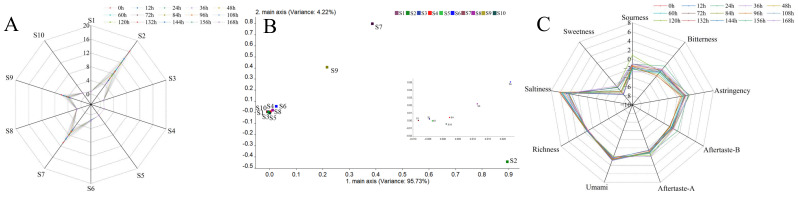
Radar chart (**A**) of response values and loadings analysis (**B**) of sensors in E-nose; radar chart of response values of taste sensors for freshly squeezed strawberry juice during cold storage (**C**).

The activity of microorganisms causes the spoilage of freshly squeezed strawberry juice. The coordination and balance of flavor and taste were broken by reducing the sugar consumption and the generation of metabolites, deteriorating the appearance, taste and nutrition of freshly squeezed strawberry juice and reducing its economic value. An E-nose consisting of 10 sensors can detect the overall aroma characteristics of freshly squeezed strawberry juice during storage. Response values of volatile organic compounds in freshly squeezed strawberry juice at different storage times are shown in the radar chart (Figure 2A).

With the extension of storage time, the response values became lower, indicating that the intensity of VOCs decreased. Loadings analysis, characterizing the contribution rate of sensors to the overall flavor characteristics, is shown in Figure 2B with the variance of PC1 reaching 95.73% and the variance of PC2 reaching 4.22%. S2, S7 and S9 contributed greatly to the first and second principal components, which are in accordance with Liu et al. [42]. Results showed that the aroma characteristics of freshly squeezed strawberry juice consisted of nitrogen oxides, inorganic sulfides and aromatic components (Table 2).

ANOVA was performed in SPSS 18.0 to explore the significance of the response values of 10 sensors. The sensor which could distinguish the most difference between the aroma characteristics of the freshly squeezed strawberry juice stored at 4 ± 1 °C for different times could be selected to fit the growth curve of total bacterial count [31]. As shown in Table 3, sensor S7 could distinguish the aroma characteristics of freshly squeezed strawberry juice samples at 12 time points in storage, while other sensors distinguished less. Freshly squeezed strawberry juice samples stored at 7 time points were distinguished by sensor S1, sensor S8 and sensor S10, and 9 time points by sensor S2 and sensor S9. Sensor S3 and sensor S6 could distinguish freshly squeezed strawberry juice stored at 8 time points. Sensor S4 and sensor S5 could discriminate 6 and 10 time points respectively.

In order to verify the correlation between the response values of sensors in E-nose and the total bacterial count, a Pearson correlation analysis was performed. As shown in Table 4, there was negative correlation (with a coefficient of −0.937) between response values of S7 and the total bacterial count, indicating that inorganic sulfides contributed more for aroma characteristic in freshly squeezed strawberry juice. It was closely related to the metabolites produced by microbial growth.

Combined with the results of the loadings analysis, ANOVA and Pearson correlation analysis, S7 was selected to fit the growth curve of total bacterial count in freshly squeezed strawberry juice during cold storage.

#### 3.2.2. Growth Simulation of Total Bacterial Count in Freshly Squeezed Strawberry Juice by an E-Nose Sensor S7

Response values of S7 were used to fit the growth curve of total bacterial count in the freshly squeezed strawberry juice during cold storage. The fitting curves are shown in Figure 1 (B1–B4). Equation and parameters are shown in Table 5. Lag time in Modified Gompertz and Logistic models was longer than in Huang and Baranyi models. R^2^ of training dataset ranged from 0.890 to 0.922, and from 0.893–0.923 in the testing dataset, both with the lower RMSE values. The correlation analysis between the fitting results and the total bacterial counts, as estimated by plate counting methods of plate agar, showed that correlation coefficients of these four models were 0.955, 0.826, 0.984 and 0.995, respectively, which could accurately simulate and predict the growth stage of total bacterial count of freshly squeezed strawberry juice during cold storage. The Huang model had the best fitting-efficiency by using S7 of the E-nose.

### 3.3. E-Tongue Testing Results

#### 3.3.1. Taste Sensor Selection

Taste features of freshly squeezed strawberry juice are shown in Figure 2C. Sourness in freshly squeezed strawberry juice was the highest at 120 h, while bitterness was high at 156 h. Pearson correlation analysis was performed between the total bacterial count from plate agar and the six basic tastes (freshness, saltiness, sourness, bitterness, astringency and sweetness) and aftertastes A and B (Table 6).

Bitterness, umami and saltiness were negatively correlated with the logarithm of total bacterial count. There was a very significant positive correlation (a coefficient of 0.772) between the response value of sweetness and total bacterial count. So, it’s feasible to simulate the growth of bacteria in freshly squeezed strawberry juice during cold storage by E-tongue.

#### 3.3.2. Growth Simulation of Total Bacterial Count in Freshly Squeezed Strawberry Juice by Sweetness Sensor

Sweetness was used to fit the growth curve of the total bacterial count in the freshly squeezed strawberry juice during cold storage shown in Figure 1(C1–C4). Equation and parameters are shown in Table 7. Lag time in logistic and Baranyi models was longer than in Modified Gompertz and Huang models, which was in accordance with the results of total bacterial count on plate agar. R^2^ of training dataset ranged from 0.861 to 0.875, and from 0.873–0.885 in the testing dataset. The correlation analysis between the fitting results and the estimated results of total bacterial count showed that correlation coefficients of these four models were 0.976, 0.954, 0.970 and 0.999, respectively, which could accurately simulate and predict the growth of total bacterial count during the cold storage of freshly squeezed strawberry juice. The fitting result of the Baranyi model by sweetness was closest to the total bacterial count on plate agar.

### 3.4. Fusion Results of E-Nose and E-Tongue

#### 3.4.1. Principal Component Scores of All Sensors in E-Nose and E-Tongue

**Figure 3:**
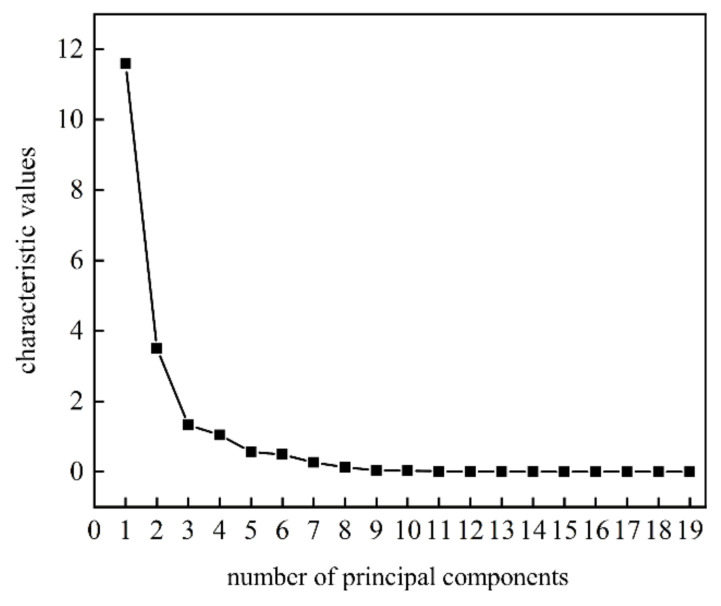
The gravel map of the principal components scores to the fusion of E-nose and E-tongue for freshly squeezed strawberry juice during cold storage.

Growth of total bacterial count in freshly squeezed strawberry juice during cold storage could cause obvious deterioration in both taste and flavor. In order to characterize the growth curve of total bacterial count more comprehensively, the principal component score method was used to fuse the response signals of E-nose and E-tongue. Combined with the gravel map (Figure 3), four principal components to maximize the information of E-nose and E-tongue were extracted with the total variations at 91.903%.

Scores of the first four principal components are shown in Table 8. With the extension of storage time, there was a downward trend on scores. Because of the negative correlation, the inverses of scores in freshly squeezed strawberry juice were used to fit the growth curve.

#### 3.4.2. Growth Simulation of Total Bacterial Count in Freshly Squeezed Strawberry Juice by Scores of the First Four Principal Components

The fitting results are shown in Figure 1(D1–D4), and the model parameters are shown in Table 9. R^2^ ranged from 0.954 to 0.964, among which the Baranyi model had the highest accuracy. The lag time obtained by the Logistic model was 75.67 h, while only 0.96 h by the Huang model. Correlation analyses were performed between the fitting results of principal components scores and the total bacterial count on plate agar with correlation coefficients ranging from 0.944 to 0.997. Compared with the fitting results of the growth curve using an E-nose or E-tongue system only, the fitting results of principal components scores were closer to the real growth stages of total bacterial in freshly squeezed strawberry juice during cold storage.

## 4. Conclusions

Gas and taste sensors were applied to predict the growth of bacteria based on changes in the odor and taste features of freshly squeezed strawberry juice during cold storage. For the E-nose, sensor S7 was selected to fit the growth curve of total bacterial count, with R^2^ values ranging from 0.890 to 0.923 and RMSE values from 0.370 to 0.478. For the E-tongue, sweetness was selected to simulate the growth curve of total bacterial count, with R^2^ values ranging from 0.861 to 0.885 and RMSE values from 0.290 to 0.364. The growth stage fitted by sweetness was closer to the fitting results of log(CFU/mL). Comparing the fitting results of traditional plate-counting estimates of total bacteria, correlation coefficients of these four models by S7 in E-nose ranged from 0.826–0.995, which were lower than those by sweetness in E-tongue (0.954–0.999). The fusion of the two systems, E-nose and E-tongue, was performed by principal components scores. Four principal components were extracted from E-nose and E-tongue to simulate the growth of microorganisms with a variance of 91.903%. R^2^ was improved to 0.954–0.964, and the correlation coefficients ranged from 0.944 to 0.997. The results indicated that microbial contamination was related to the changes in the aroma and taste features of freshly squeezed strawberry juice during cold storage. E-nose and E-tongue readings could be applied to simulate and predict the growth of bacteria in freshly squeezed strawberry juice during cold storage. Gas and taste sensors have potential applications as tools for the predictive microbiology of fruit juice.

## Figures and Tables

**Table 1:** Different kinetic-fitting models and parameters of the total bacterial count by traditional plate counting in freshly squeezed strawberry juice during cold storage.

Models	***λ*** **(h)**	***μ_max_*** **(h^−1^)**	***R* ^2^**	**RMSE**	***N*_0_** **(LogCFU/mL)**	**N_max_ (LogCFU/mL)**
Modified Gompertz	45.000	0.013	0.953	0.132	3.878	6.117
Logistic	108.400	0.062	0.971	0.103	3.992	5.341
Huang	55.180	0.015	0.944	0.143	3.975	6.558
Baranyi	97.850	0.064	0.971	0.104	3.996	5.337

**Table 2:** The description of E-nose sensor performance.

Number	Sensors	Sensitivity
S1	W1C	Sensitive to aromatics, benzene
S2	W5S	High sensitivity, especially nitrogen oxides
S3	W3C	Ammonia, aromatic components
S4	W6S	Mainly selective for hydrogen
S5	W5C	Sensitive to alkane aromatic components
S6	W1S	Sensitive to short-chain alkanes, methane
S7	W1W	Sensitive to inorganic sulfides
S8	W2S	Sensitive to alcohol, ether, aldehydes and ketones
S9	W2W	Aromatic components, organic sulfides
S10	W3S	Sensitive to long-chain alkanes

Note: Reprinted/Adapted with permission from Ref. [43]. 2018, Fan J. et al.

**Table 3:** ANOVA of the response values in E-nose of freshly squeezed strawberry juice during cold storage.

	S1	S2	S3	S4	S5	S6	S7	S8	S9	S10
0 h	0.754 ± 0.020 ^g^	16.176 ± 2.912 ^a^	0.881 ± 0.011 ^h^	1.065 ± 0.007 ^a^	0.915 ± 0.005 ^j^	1.776 ± 0.093 ^a^	10.719 ± 0.942 ^a^	1.358 ± 0.039 ^ab^	6.022 ± 0.433 ^a^	1.113 ± 0.005 ^cd^
12 h	0.772 ± 0.014 ^f^	13.414 ± 1.918 ^b^	0.891 ± 0.007 ^g^	1.051 ± 0.006 ^b^	0.924 ± 0.005 h^i^	1.670 ± 0.071 ^b^	9.445 ± 0.995 ^b^	1.337 ± 0.035 ^ab^	5.334 ± 0.491 ^b^	1.116 ± 0.005 ^bc^
24 h	0.769 ± 0.013 ^f^	12.626 ± 2.205 ^b^	0.888 ± 0.008 ^g^	1.047 ± 0.004 ^c^	0.922 ± 0.005 ^i^	1.633 ± 0.035 ^bc^	9.115 ± 0.865 ^bc^	1.335 ± 0.019 ^ab^	5.124 ± 0.375 ^b^	1.113 ± 0.004 ^cd^
36 h	0.783 ± 0.017 ^e^	10.269 ± 3.127 ^d^	0.898 ± 0.007 ^f^	1.051 ± 0.009 ^b^	0.929 ± 0.005 ^g^	1.651 ± 0.065 ^bc^	8.733 ± 1.298 ^c^	1.345 ± 0.034 ^a^	4.816 ± 0.561 ^c^	1.130 ± 0.008 ^a^
48 h	0.787 ± 0.008 ^e^	11.444 ± 1.352 ^c^	0.896 ± 0.004 ^f^	1.048 ± 0.008 ^bc^	0.926 ± 0.003 ^h^	1.623 ± 0.033 ^c^	7.937 ± 1.016 ^d^	1.317 ± 0.020 ^c^	4.557 ± 0.401 ^d^	1.127 ± 0.012 ^a^
60 h	0.789 ± 0.014 ^e^	9.672 ± 1.278 ^de^	0.899 ± 0.007 ^f^	1.047 ± 0.005 ^c^	0.930 ± 0.005 ^g^	1.639 ± 0.064 ^bc^	7.676 ± 0.992 ^def^	1.328 ± 0.033 ^bc^	4.451 ± 0.395 ^d^	1.127 ± 0.009 ^a^
72 h	0.804 ± 0.012 ^d^	8.997 ± 1.217 ^ef^	0.907 ± 0.007 ^e^	1.045 ± 0.005 ^c^	0.934 ± 0.004 ^f^	1.566 ± 0.034 ^de^	7.731 ± 0.610 ^de^	1.293 ± 0.019 ^d^	4.356 ± 0.282 ^d^	1.127 ± 0.008 ^a^
84 h	0.826 ± 0.010 ^b^	8.170 ± 0.705 f^g^	0.919 ± 0.005 ^b^	1.041 ± 0.013 ^d^	0.943 ± 0.003 ^c^	1.492 ± 0.040 ^g^	7.243 ± 0.656 ^fg^	1.244 ± 0.021 ^f^	4.073 ± 0.283 ^e^	1.108 ± 0.004 ^ef^
96 h	0.806 ± 0.013 ^cd^	8.627 ± 1.105 ^f^	0.908 ± 0.007 ^de^	1.04 ± 0.003 ^de^	0.936 ± 0.004 ^ef^	1.575 ± 0.040 ^d^	7.318 ± 0.550 ^efg^	1.293 ± 0.023 ^d^	4.129 ± 0.258 ^e^	1.125 ± 0.007 ^a^
108 h	0.810 ± 0.012 ^cd^	8.220 ± 1.179 ^fg^	0.910 ± 0.006 ^de^	1.037 ± 0.002 ^ef^	0.938 ± 0.004 ^de^	1.567 ± 0.033 ^de^	7.171 ± 0.586 ^gh^	1.284 ± 0.018 ^de^	4.019 ± 0.248 ^ef^	1.119 ± 0.007 ^b^
120 h	0.819 ± 0.022 ^bc^	7.406 ± 1.410 ^g^	0.915 ± 0.013 ^cd^	1.037 ± 0.003 ^ef^	0.941 ± 0.008 ^d^	1.536 ± 0.055 ^def^	6.778 ± 0.382 ^hi^	1.270 ± 0.028 ^e^	3.851 ± 0.193 ^fg^	1.115 ± 0.008 ^bc^
132 h	0.822 ± 0.011 ^b^	6.375 ± 0.805 ^h^	0.916 ± 0.006 ^bc^	1.034 ± 0.003 ^fg^	0.943 ± 0.004 ^c^	1.535 ± 0.030 ^ef^	6.673 ± 0.496 ^ij^	1.271 ± 0.015 ^e^	3.802 ± 0.224 ^g^	1.111 ± 0.007 ^de^
144 h	0.851 ± 0.013 ^a^	5.727 ± 0.651 ^h^	0.931 ± 0.006 ^a^	1.034 ± 0.003 ^fg^	0.954 ± 0.004 ^b^	1.455 ± 0.043 ^h^	6.245 ± 0.578 ^jk^	1.221 ± 0.022 ^g^	3.590 ± 0.272 ^h^	1.105 ± 0.006 ^fg^
156 h	0.847 ± 0.033 ^a^	4.738 ± 1.115 ^i^	0.933 ± 0.015 ^a^	1.035 ± 0.005 ^fg^	0.958 ± 0.008 ^a^	1.522 ± 0.083 ^f^	5.947 ± 0.756 ^kl^	1.241 ± 0.042 ^f^	3.387 ± 0.310 ^i^	1.101 ± 0.008 ^g^
168 h	0.842 ± 0.029 ^a^	4.737 ± 0.989 ^i^	0.931 ± 0.015 ^a^	1.033 ± 0.004 ^g^	0.956 ± 0.009 ^ab^	1.527 ± 0.093 ^f^	5.724 ± 0.604 ^l^	1.254 ± 0.046 ^f^	3.215 ± 0.287 ^i^	1.106 ± 0.006 ^f^

Note: Values are means ± standard deviation; different lower case letters mean statistically significant (*p* < 0.05).

**Table 4:** Pearson correlation analysis between the E-nose response values and the total bacterial count of freshly squeezed strawberry juice during cold storage.

	S1	S2	S3	S4	S5	S6	S7	S8	S9	S10	Log(CFU/mL)
S1	1										
S2	−0.960 **	1									
S3	0.996 **	−0.960 **	1								
S4	−0.903 **	0.943 **	−0.887 **	1							
S5	0.986 **	−0.959 **	0.996 **	−0.889 **	1						
S6	−0.935 **	0.903 **	−0.908 **	0.921 **	−0.885 **	1					
S7	−0.951 **	0.981 **	−0.943 **	0.950 **	−0.934 **	0.906 **	1				
S8	−0.972 **	0.885 **	−0.954 **	0.869 **	−0.934 **	0.952 **	0.890 **	1			
S9	−0.954 **	0.987 **	−0.950 **	0.953 **	−0.942 **	0.909 **	0.998 **	0.891 **	1		
S10	−0.569 *	0.415	−0.585 *	0.434	−0.615 *	0.459	0.389	0.635 *	0.392	1	
Log(CFU/mL)	0.847 **	−0.906 **	0.823 **	−0.883 **	0.791 **	−0.875 **	−0.937 **	−0.806 **	−0.934 **	−0.142	1

Note: * means statistically significant (*p* < 0.05); ** means statistically extremely significant (*p* < 0.01).

**Table 5:** Different kinetic-fitting models and parameters of E-nose sensor S7 in freshly squeezed strawberry juice during cold storage.

Models	*λ* (h)	***μ_max_*** **(h^−1^)**	Training		Testing		*r*
			*R_c_* ^2^	*RMSE_c_*	*R_p_* ^2^	*RMSE_p_*	
Modified Gompertz	10.000	0.031	0.890	0.458	0.893	0.478	0.955
Logistic	22.300	0.027	0.914	0.405	0.919	0.401	0.826
Huang	2.400	0.030	0.920	0.407	0.920	0.414	0.984
Baranyi	2.900	0.030	0.922	0.370	0.923	0.407	0.995

**Table 6:** Pearson correlation analysis between the E-tongue response values and the total bacterial count of freshly squeezed strawberry juice during cold storage.

	Sourness	Bitterness	Astringency	Aftertaste-B	Aftertaste-A	Umami	Richness	Saltiness	Sweetness	Log(CFU/mL)
Sourness	1									
Bitterness	0.292	1								
Astringency	0.667 **	0.764 **	1							
Aftertaste-B	0.342	0.931 **	0.702 **	1						
Aftertaste-A	0.577 *	0.405	0.666 **	0.512	1					
Umami	−0.812 **	−0.511	−0.774 **	−0.514 *	−0.464	1				
Richness	0.032	−0.092	0.106	0.095	0.121	−0.131	1			
Saltiness	−0.622 *	−0.722 **	−0.904 **	−0.622 *	−0.372	0.854 **	−0.076	1		
Sweetness	0.533 *	0.323	0.668 **	0.302	0.325	−0.867 **	0.304	−0.777 **	1	
Log(CFU/mL)	0.243	−0.044	0.219	0.092	0.166	−0.578 *	0.641 **	−0.349	0.772 **	1

Note: * means statistically significant (*p* < 0.05); ** means statistically extremely significant (*p* < 0.01).

**Table 7:** Different kinetic-fitting models and parameters of sweetness by E-tongue in freshly squeezed strawberry juice during cold storage.

Models	*λ* (h)	***μ_max_*** **(h^−1^)**	Training		Testing		*r*
			*R_c_* ^2^	*RMSE_c_*	*R_p_* ^2^	*RMSE_p_*	
Modified Gompertz	23.240	0.020	0.863	0.345	0.873	0.310	0.976
Logistic	83.590	0.053	0.875	0.345	0.885	0.295	0.954
Huang	38.070	0.030	0.861	0.364	0.881	0.290	0.970
Baranyi	69.820	0.065	0.874	0.331	0.884	0.296	0.999

**Table 8:** Principal components scores of the response values of E-nose and E-tongue for freshly squeezed strawberry juice during cold storage.

Time/h	PC1	PC2	PC3	PC4	Score
	60.98%	18.43%	6.99%	5.51%	
0	5.50	3.34	−0.51	−1.84	3.83
12	3.73	1.64	−1.07	0.00	2.50
24	3.38	0.50	−0.65	0.64	2.14
36	3.16	0.17	−0.37	1.67	2.02
48	1.94	0.50	0.80	0.85	1.37
60	2.13	−0.50	0.39	0.18	1.25
72	1.33	−1.87	1.32	0.34	0.58
84	−0.53	−2.28	−0.66	−0.44	−0.81
96	0.90	−1.68	0.94	−0.45	0.28
108	−2.08	−0.03	1.16	0.09	−1.19
120	−3.31	1.41	2.39	−0.37	−1.61
132	−2.42	−0.53	−0.83	−1.34	−1.70
144	−3.59	−2.38	−1.32	−1.35	−2.79
156	−5.24	0.81	−1.80	1.72	−3.08
168	−4.89	0.90	0.20	0.29	−2.79

**Table 9:** Different kinetic-fitting models and parameters from scores of PC1–PC4 extracted by E-nose and E-tongue sensors for freshly squeezed strawberry juice during cold storage.

Models	***λ*** **(h)**	***μ_max_*** **(h^−1^)**	***R* ^2^**	***RMSE***	***r***
Modified Gompertz	9.240	0.050	0.954	0.505	0.968
Logistic	75.670	0.022	0.961	0.462	0.944
Huang	0.960	0.045	0.963	0.450	0.993
Baranyi	8.897	0.045	0.964	0.464	0.997

## Data Availability

No data available.
